# Oral GAD65-*L. lactis* Vaccine Halts Diabetes Progression in NOD Mice by Orchestrating Gut Microbiota–Metabolite Crosstalk and Fostering Intestinal Immunoregulation

**DOI:** 10.3390/microorganisms14010176

**Published:** 2026-01-13

**Authors:** Shihan Zhang, Xinyi Wang, Chunli Ma, Tianyu Liu, Qingji Qin, Jiandong Shi, Meini Wu, Jing Sun, Yunzhang Hu

**Affiliations:** 1 Institute of Medical Biology, Chinese Academy of Medical Sciences and Peking Union Medical College, Kunming 650118, China; zsh15226927851@163.com (S.Z.); wxyvicky@yeah.net (X.W.); 20191167@kmmu.edu.cn (C.M.); kingofthemountain2022@gmail.com (T.L.); changeqin@outlook.com (Q.Q.); shijiandong@imbcams.com.cn (J.S.); nini5440@163.com (M.W.); 2 School of Food and Pharmacy, Yuxi Vocational and Technical College, Yuxi 653100, China; 3 Kunming Medical University, Kunming 650031, China; 4 Department of Medical Laboratory, The Third People’s Hospital of Honghe, Gejiu 661100, China

**Keywords:** T1D, GAD65-*L. lactis* vaccine, 16S rRNA, metabolomics, immunomodulation

## Abstract

This study successfully developed an oral vaccine for Type 1 Diabetes utilizing recombinant *Lactococcus lactis* expressing the GAD65 autoantigen. We conducted an in-depth investigation into its protective mechanisms in NOD mice, with a particular focus on its effects on the gut microbiota and metabolome. The administration of the GAD65-*L. lactis* vaccine resulted in a significant delay in diabetes onset and the preservation of pancreatic function. Our analyses revealed notable alterations in the gut microbial ecosystem, enhancing its diversity and the abundance of beneficial bacteria. Metabolomic profiling indicated time-dependent changes in metabolic pathways, with a marked enrichment of pyrimidine metabolism at 16 weeks and arachidonic acid metabolism at 24 weeks after vaccination by both GAD65-*L. lactis* and NZ9000-*L. lactis*. Integrated correlation analysis identified specific microbiota–metabolite interactions, including associations between *Ruminiclostridium* and lipid species in the GAD65-*L. lactis* group. These modifications in the microbial community and metabolic landscape were accompanied by enhanced immunoregulatory responses in intestinal LPLs, including expanded Treg populations and suppressed CD8^+^ T cells, a rising trend in IL-10-producing naive dendritic cells, and increased concentrations of TGF-β.

## 1. Introduction

T1D is an autoimmune disease characterized by a breakdown in immune self-tolerance, leading to autoimmune destruction of pancreatic β-cells. This results in compromised insulin secretion, dysregulated glucose metabolism, and progressive hyperglycemia [1]. The pathogenesis of T1D involves a complex interplay between genetic predisposition and environmental triggers [2,3,4,5]. Current treatment for T1D primarily relies on exogenous insulin replacement therapy [6]; however, this approach fails to effectively maintain glycemic control. In contrast, the development of T1D vaccines aims to modulate the patient’s immune system to prevent and mitigate disease progression, thereby improving patients’ quality of life and alleviating the burden on healthcare resources [7]. In pursuit of a safer and potentially disease-modifying strategy for Type 1 Diabetes (T1D), we have developed an oral immunomodulatory approach. Specifically, we engineered a recombinant Lactococcus lactis (*L. lactis*) vaccine that expresses the T1D antigen GAD65. This was achieved by cloning the GAD65 gene into the pNZ8148 vector and expressing it in *L. lactis* NZ9000. The purpose of this vaccine is to induce regulatory T cell (Treg) populations and restore immune tolerance in the host [8].

*L. lactis* is a common gut microorganism generally regarded as safe for humans and animals, with a long history of safe use in fermented foods and well-established technological applications [9]. This makes it a promising and relatively safe candidate for oral vaccine delivery. In addition to its established safety profile, *L. lactis* offers unique advantages as a probiotic vehicle for oral immunization. It can enhance oral tolerance by modulating the gut microbiota and strengthening intestinal barrier function [10]. Specifically, *L. lactis* contributes to a healthier gut environment through several mechanisms: (1) It produces acidic metabolites, such as lactic and acetic acid, which lower intestinal pH, inhibit the growth of pathogens, and create conditions conducive to the proliferation of beneficial bacteria [11]; (2) It aids in increasing microbial diversity and stabilizing the gut microbial community [12]; and (3) Through the fermentation of dietary fibers, it generates short-chain fatty acids (SCFAs) [13], which serve as energy sources, regulate immune responses, and further modulate the intestinal environment. Despite these promising attributes, substantial research gaps persist. Existing studies have predominantly concentrated on the general safety and specific probiotic functions of *L. lactis.* However, there remains a paucity of comprehensive knowledge concerning its long-term stability and efficacy as an oral vaccine vector in vivo. Critical questions that remain unanswered include the exact mechanisms through which it interacts with the host immune system, the manner in which it modulates the gut microenvironment to promote antigen-specific immune tolerance, and its systemic impact on the microbial community structure and host metabolism in vaccinated individuals.

In addition to established risk factors such as genetic predisposition and environmental influences, the roles of gut microbiota and metabolome dynamics in the pathogenesis and progression of diabetes have garnered growing scientific attention [14,15]. The gut microbiota can precisely regulate the mucosal immune system [16]. A healthy gut microbiota contributes to maintaining immune homeostasis. Under physiological conditions, the gut microbial community establishes a mutually beneficial relationship with the host; conversely, microbial dysbiosis may induce alterations in intestinal epithelial permeability or metabolic processes, thereby impairing the functionality of gut mucosal immune cells [17]. Thus, the gut microbiota modulates metabolic pathways, which in turn influence the integrity of the intestinal mucosal immune barrier [18]. Within this context, metabolomics is increasingly recognized as an essential tool for evaluating the functional state of the gut microbiota. By identifying changes in the concentrations and varieties of bacterial metabolites—such as amino acids, lipids, and carbohydrates—researchers can elucidate functional connections between microbial metabolic activity and immune responses elicited by vaccines [19]. The integration of 16S rRNA sequencing and metabolomics technologies allows comprehensive investigation into the characteristics of gut microbiota and metabolomes in diabetic individuals, elucidating the protective effects mediated by NZ9000 as a vaccine vector. This approach provides novel insights for T1D prevention and therapeutic strategies.

This study randomly divided 6-week-old NOD mice into three groups: a PBS control group, a GAD65-*L. lactis* group, and an NZ9000-*L. lactis* group. Blood glucose, body weight, and survival rates were monitored regularly. At the endpoint, lymphocytes from the intestine and spleen were collected to analyze the proportions of Treg cells, CD8^+^ T cells, and CD80^+^CD11c^+^ dendritic cells (DCs). Additionally, fecal samples were collected at weeks 7, 16, 24 and 32 for microbiome and metabolome analyses. The results demonstrated that the GAD65-*L. lactis* formulation effectively reduced mortality, delayed the onset of diabetes, and increased C-peptide expression levels, thereby preserving islet function. Furthermore, it mainly inhibited LPLs CD8^+^ T lymphocytes and the proliferation of mature DCs, while showing an increasing trend in IL-10-secreting naive DCs, which subsequently induced the proliferation of Tregs; concurrently, elevated levels of TGF-β were detected, indicating that this vaccine induced a tolerance-promoting environment in the intestine. Following vaccination, the phyla *Bacteroidota* and *Firmicutes* emerged as predominant, with *Muribaculaceae* and *Alistipes* as the principal genera. At 16 weeks, both treatment groups exhibited enrichment in pyrimidine metabolism; however, GAD65-*L. lactis* demonstrated a more pronounced enrichment in linoleic acid metabolism. By 24 weeks, arachidonic acid metabolism was significantly enriched across all groups. An integrated analysis revealed treatment-specific correlations between the microbiota and metabolites, such as the association of *Ruminiclostridium* with certain lipids in the GAD65-*L. lactis* group at 16 weeks, and various genus–metabolite shifts in the NZ9000-*L. lactis* groups.

Our study demonstrates that oral GAD65-*L. lactis* induces antigen-specific immune tolerance and modulates the gut microbiome–metabolome axis, effectively delaying diabetes in NOD mice. These findings highlight a novel, gut-targeted treatment strategy for combating autoimmune diabetes.

## 2. Materials and Methods

### 2.1. Animals, Experimental Design and Sampling

All animal experiments were approved and conducted in accordance with the requirements of the Animal Management and Use Committee of the Institute of Medical Biology, Chinese Academy of Medical Sciences and Peking Union Medical College. Mice were housed under a 12 h light–dark cycle, at a temperature of 22 ± 2 °C and humidity of 50–60%, with free access to food and water.

In the NOD mouse model, female exhibit higher and more stable rates of spontaneous T1D onset, along with faster disease progression [20]. Therefore, to conduct the experiment, Six-week-old female NOD mice were randomly divided into three groups (14 mice per group). Following a one-week acclimatization period, the mice received oral vaccine gavage according to the doses specified in Table 1. As illustrated in Figure S1A, the first phase consisted of seven consecutive days of oral administration, followed by a second seven-day phase after a two-week interval. Upon completion of the immunization protocol, 3 mice per group were randomly selected for euthanasia to assess *Lactococcus lactis* intestinal colonization. At two weeks post-immunization, 5 mice per group underwent euthanasia with sample collection to evaluate vaccine immunogenicity. The remaining mice (6 mice per group) were maintained under standard housing conditions until 32 weeks of age followed by euthanasia and tissue collection.

From week 6 to week 32, body weight and blood glucose levels were monitored weekly. For glucose measurements, mice were fasted for 6 h prior to testing. Body weight was recorded using an electronic scale (Shunyuhengping Science, MP2002, Shanghai, China), and venous blood samples were collected via tail clipping (1–2 mm) for blood glucose testing (OneTouch VerioVue, Chesterbrook, PA, USA). Mice exhibiting blood glucose levels exceeding 10.3 mmol/L in two consecutive measurements were classified as hyperglycemic, indicating the onset of T1D.

### 2.2. Intestinal Colonization Assay

Twelve hours after the final immunization, intestinal contents were collected from the mice and serially diluted in sterile PBS from 10^−1^ to 10^−10^ to assess the survival and colonization capacity of *L. lactis* in the gut. Diluted suspensions were plated onto solid GM medium containing chloramphenicol (20 μg/mL) and incubated anaerobically at 30 °C.

### 2.3. Fecal Sample Collection

Fecal samples were collected from NOD mice at 7, 16, 24 and 32 weeks of age. Mice were monitored for defecation, and fresh fecal pellets were collected under aseptic conditions using the tail-lifting method to avoid contamination. Collected samples were immediately transferred to liquid nitrogen-prechilled EP tubes, flash-frozen in liquid nitrogen to preserve microbial viability and metabolome integrity, and stored at −80 °C until analysis. The collection time, date, and corresponding mouse identifiers were meticulously documented to ensure traceability during downstream multi-omics profiling.

### 2.4. Detection of Target Biomolecules by ELISA

Blood samples (≥100 μL per mouse) were obtained from NOD mice under isoflurane anesthesia through retro-orbital bleeding. All blood samples were incubated at 37 °C for 1 h to allow clotting, followed by centrifugation at 3000× *g* for 15 min to separate serum.

The 96-well flat-bottom plates were coated with 100 μL/well of 0.1 μg/mL recombinant human GAD65 protein (huGAD65, Abcam, Cambridge, MA, USA) in coating buffer and incubated overnight at 4 °C. After blocking with 1% BSA at 37 °C for 1 h, plates underwent four washes with wash buffer. Then, mice fecal diluent samples were serially diluted in two-fold dilutions in blocking buffer (initial concentration at 1:10) and incubated for 1 h at 37 °C, followed by four washes. Subsequently, HRP-labeled goat anti-mouse IgA (Abcam, USA; 1:1000 dilution) was added and incubated at 37 °C for 1 h. Following four additional washes, 100 μL of ABTS HRP substrate was added and allowed to develop in the dark for 15 min. The reaction was terminated with 25 μL of ABTS peroxidase stop solution. All ELISA detection reagents were sourced from the Anti-Mouse ABTS^®^ System ELISA Kit (KPL Protein Detector™, Gaithersburg, MD, USA).

The concentration of C-Peptide was detected using a double antibody sandwich enzyme-linked immunosorbent assay kit (Sangon, D721064-0096, Shanghai, China). The lyophilized powder was diluted to 10 ng/mL using the standard diluent. The samples were analyzed by enzyme-linked immunosorbent assay (OD = 450 nm), and the C-Peptide concentration was calculated based on the standard curve.

Serum TGF-β levels were quantified using the Human/Mouse TGF-β Uncoated ELISA Kit (Thermo Scientific, Catalog No. 88-8350-88, Waltham, MA, USA) according to the manufacturer’s protocol. For quality assurance, standard curves were generated to verify sample concentrations within the linear detection range.

Optical density was measured at 450 nm using an ELISA plate reader (UVP, LLC, Upland, CA, USA) with all samples analyzed in triplicate.

### 2.5. Flow Cytometry Assay

#### 2.5.1. Splenic Lymphocyte Isolation

Mice splenocytes were harvested at weeks 14 and 32. The spleen was excised and placed on sterile gauze. After gentle grinding, the gauze was rinsed with 5 mL of serum-free RPMI-1640 medium (VivaCell, C3010-0500, Shanghai, China) to collect the cell suspension. The suspension was centrifuged at 500× *g* for 5 min (Centrifuge: Hitachi CT15RE, Tokyo, Japan), and the supernatant was discarded. The pellet was resuspended in 3 mL of pre-warmed ACK lysing buffer (eBioscience 00-4300-54, Carlsbad, CA, USA) and incubated at room temperature for 5 min. The reaction was terminated by adding 5 mL of complete RPMI-1640 medium (1640 medium containing 10% FBS (VivaCell, C04001-050X, China)). After centrifugation at 500× *g* for 5 min, the supernatant was discarded, and the washing step was repeated once. The cell pellet was resuspended in complete RPMI-1640 medium and adjusted to a concentration of 1 × 10^6^ cells/mL for subsequent use.

#### 2.5.2. Intestinal Lamina Propria Lymphocyte Isolation

Mice Intestinal Lamina Propria Lymphocytes (LPLs) were harvested at week 14 and 32. Small intestinal tissues were placed in a Petri dish containing PBS (VivaCell, C3580-0500, China). Mesenteric lymph nodes and adipose tissues were removed. The intestines were longitudinally opened, rinsed three times with PBS, and cut into 2 cm segments. Segments were transferred to a 50 mL tube containing 5 mL of dissociation buffer and incubated at 37 °C for 15 min with shaking at 250 rpm. Tissues were vortexed for 30 s and filtered through sterile gauze; the filtrate was discarded, and retained tissues underwent a second digestion. Tissues were transferred to a new tube with 5 mL digestion buffer and shaken at 37 °C (250 rpm) for 45 min. After vortexing (30 s) and gauze filtration, the filtrate was collected into a 15 mL tube and centrifuged at 400× *g* (4 °C) for 10 min. Then the supernatant was discarded. 4 mL of 80% Percoll (Cytiva 17-0891-01, Uppsala, Sweden) was added to the 15 mL tube, and the cell pellet was resuspended in 8 mL of 40% Percoll and carefully layered onto the 80% Percoll solution. Gradient separation was performed by centrifugation at 500× *g* (20 °C) for 20 min. The top 5 mL of supernatant was discarded, and the interphase cell layer was collected. Cells were diluted with PBS to 15 mL, mixed gently, and centrifuged at 400× *g* (4 °C) for 8 min. The pellet was resuspended in an appropriate volume of complete RPMI-1640 medium for subsequent use. The formulation of the dissociation buffer consists of 55 mL of Fetal Bovine Serum, 100 μL of 2 mM EDTA, 100 μL of 1 nM DTT (Gibco, 16000044, Grand Island, NY, USA), and 83 mL of 1 M HEPES Buffer (VivaCell, C3544-0100, China). The digestion buffer, on the other hand, is composed of 2.5 mL of Fetal Bovine Serum, 1 mL of a solution containing 1.5 g Collagenase Type VIII (Sigma, C2139, St. Louis, MO, USA), 5 mL of DNase I (Invitrogen, 18047019, Waltham, MA, USA), and 85 mL of PBS containing calcium and magnesium ions (VivaCell, C3593-500, China).

#### 2.5.3. Flow Cytometry Staining

Lymphocytes isolated from the lamina propria and spleen were stimulated with a synthetic GAD65 peptide mixture (final concentration: 5 μg/mL) at 37 °C with 5% CO^2^ for 48 h in 96-well plates. The peptide sequences comprised: TYEIAPVFVLLEYVT, EYVTLKKMREIIGWPGGSGD, KKGAAALGIGTDSVI, ALGIGTDSVILIKCDERGK and TLEDNEERMSRLSK. The peptides were synthesized by Shanghai Anlian Peptide Co., Ltd. located in Shanghai, China. Their purity exceeded 95%. After stimulation, 100 µL of Flow Cytometry Staining Buffer was added to each well, followed by 1 µL of fluorochrome-conjugated surface marker antibody. CD8^+^ T lymphocytes: anti-mouse CD8a-PE (eBioscience, 53-6.7, 12-0081-82, USA), CFSE Cell Division Kit (eBioscience, 65-0850-84, USA). Treg cells: anti-mouse CD4-FITC (eBioscience, GK1.5, 11-0041-82, USA), anti-mouse CD25-PE (eBioscience, PC61.5, 12-0251-82, USA), anti-mouse/Rat-Foxp3-PE (eBioscience, FJK-16s, 12-5773-80A, USA). Dendritic cells: anti-mouse CD80-APC (eBioscience, 16-10A1, 17-0801-82, USA), anti-mouse CD11c-PE (eBioscience, N418, 12-0114-82, USA), anti-mouse IL10-FITC (eBioscience, JES5-16E3, 11-7101-82, USA). Cells were incubated in the dark at 4 °C for ≥30 min. After incubation, cells were washed with 1× Permeabilization Buffer (eBioscience,00-8333-56, USA) (200 µL/well) and centrifuged at 500× *g* for 5 min at room temperature. The supernatant was discarded, and this washing procedure was repeated twice. Following the final wash, cells were resuspended in 100 µL of 1× Permeabilization Buffer per well. Samples were analyzed using a CytoFLEX flow cytometer (Beckman Coulter, CytoFLEX, Brea, CA, USA). The flow data were processed using CytExpert software 2.3.1.22.

### 2.6. DNA Extraction and 16s rRNA Sequencing

Bacterial genomic DNA was extracted from mouse fecal samples using the DNeasy PowerSoil Kit (Qiagen, Valencia, CA, USA) following the manufacturer’s instructions. PCR amplification was performed with genomic DNA as the template, using barcoded primers and Takara Ex Taq polymerase (Takara Bio, Dalian, China). For bacterial diversity analysis, the V3–V4 hypervariable regions of the 16S rRNA gene were amplified with universal primers 343F and 798R. The amplicons were purified twice with AMPure XP beads (Beckman Coulter, USA) and subjected to a second round of PCR for indexing. Final amplicons were quantified using the Qubit dsDNA Assay Kit (Life Technologies, Carlsbad, CA, USA). Representative reads were taxonomically annotated with the RDP Classifier (70% confidence threshold) and analyzed via BLAST (2.15.0) against the SILVA database (version 123) and Greengenes (16S rRNA reference). Sequencing was performed on the Illumina NovaSeq platform by OE Biotech Co., Ltd. (Shanghai, China). Raw sequence data and metadata have been deposited in the NCBI Sequence Read Archive (SRA) under BioProject accession number PRJNA1254855.

### 2.7. Untargeted Metabolomics Analysis

#### 2.7.1. Sample Preparation

Approximately 60 mg of frozen feces was weighed into a 1.5 mL microcentrifuge tube, followed by addition of 20 μL of pre-cooled internal standard solution (L-2-chlorophenylalanine, 0.06 mg/mL in methanol) and 600 μL of ice-cold methanol–water extraction solvent (4:1, *v*/*v*). Two steel beads were added, and the mixture was equilibrated at −20 °C for 5 min before mechanical homogenization at 60 Hz for 2 min. After ice-bath sonication for 10 min, the sample was incubated at −20 °C for 30 min and centrifuged at 13,000 rpm (4 °C) for 10 min. A 200 μL aliquot of the supernatant was transferred and dried under a nitrogen stream. The residue was reconstituted in 300 μL of methanol–water solvent (1:4, *v*/*v*), vortex-mixed for 30 s, and sonicated in an ice bath for 3 min. Following incubation at −20 °C for 2 h and centrifugation at 13,000 rpm (4 °C) for 10 min, 150 μL of the supernatant was filtered through a 0.22 μm organic-compatible membrane and stored at −80 °C prior to analysis.

#### 2.7.2. LC-MS Analysis

Separation was performed using an ACQUITY UPLC I-Class Plus system (Waters, Milford, MA, USA) coupled to a Q Exactive HF-X high-resolution mass spectrometer. Chromatographic separation employed an ACQUITY UPLC HSS T3 column (100 mm × 2.1 mm, 1.8 μm) maintained at 45 °C. The mobile phase consisted of: A (0.1% formic acid in water) and B (0.1% formic acid in acetonitrile) at a flow rate of 0.35 mL/min, with an injection volume of 2 μL. The gradient program was as follows: 95% A (0–2 min), linear transition to 70% A at 4 min, 50% A at 8 min, 20% A at 10 min, 100% B (14–15 min), followed by column re-equilibration at 95% A (15.1–16 min).

Mass spectrometric detection utilized an electrospray ionization (ESI) source with positive/negative ionization switching, scanning over 100–1200 *m*/*z*. Resolution was set to 70,000 for full MS scans (MS1) and 17,500 for MS/MS scans (MS2). Source parameters were configured as: spray voltage +3.8 kV (positive)/−3.2 kV (negative), sheath gas 40 Arb, auxiliary gas 10 Arb, and capillary temperature 320 °C. Raw data were processed by Lu-Ming Biotech (Shanghai, China) using Compound Discoverer 3.3 software (Thermo Scientific). The raw data have been deposited in the Genome Sequence Archive (GSA) database under accession number OMIX011740.

### 2.8. Integrated Analysis

Associations between differentially abundant gut microbiota and differentially abundant metabolites were evaluated using Spearman’s rank correlation analysis in R version 4.1.2.

### 2.9. Statistical Analysis

Statistical analysis was performed using GraphPad Prism software (version 9.0, GraphPad Software, San Diego, CA, USA). One-way analysis of variance (ANOVA) was used to compare continuous variables. Categorical data were expressed as percentages. Differences between two groups were assessed using Student’s *t*-test or the Mann–Whitney U test (Wilcoxon rank-sum test). The threshold for statistical significance was set at *p* < 0.05. Differential metabolites were screened via PLS-DA and validated using a two-tailed Student’s *t*-test. The selection criteria were VIP > 1.0 and *p* < 0.05. Subsequently, these differential metabolites were annotated using the KEGG database (https://www.kegg.jp/kegg/pathway.html, accessed on 19 September 2025) for metabolic pathway analysis to identify the pathways in which they are involved.

## 3. Results

### 3.1. Colonization of Recombinant Lactococcus lactis in the Gut

In NOD mice, we observed (Figure S1B) that NZ9000 strains carrying either pNZ8148 or huGAD65 exhibited significantly enhanced survival in vivo compared to the PBS-treated control group when cultured on GM medium supplemented with antibiotics (*p* < 0.05), while there was no difference in colonization efficiency between NZ9000-*L. lactis* group and GAD65-*L. lactis* group (*p* > 0.05). The data for groups control and GAD65 (Figure S1B) are from our previous work [9].

### 3.2. The Effect of GAD65-L. lactis on Suppressed Hyperglycemia and Diabetes

**Figure 1:**
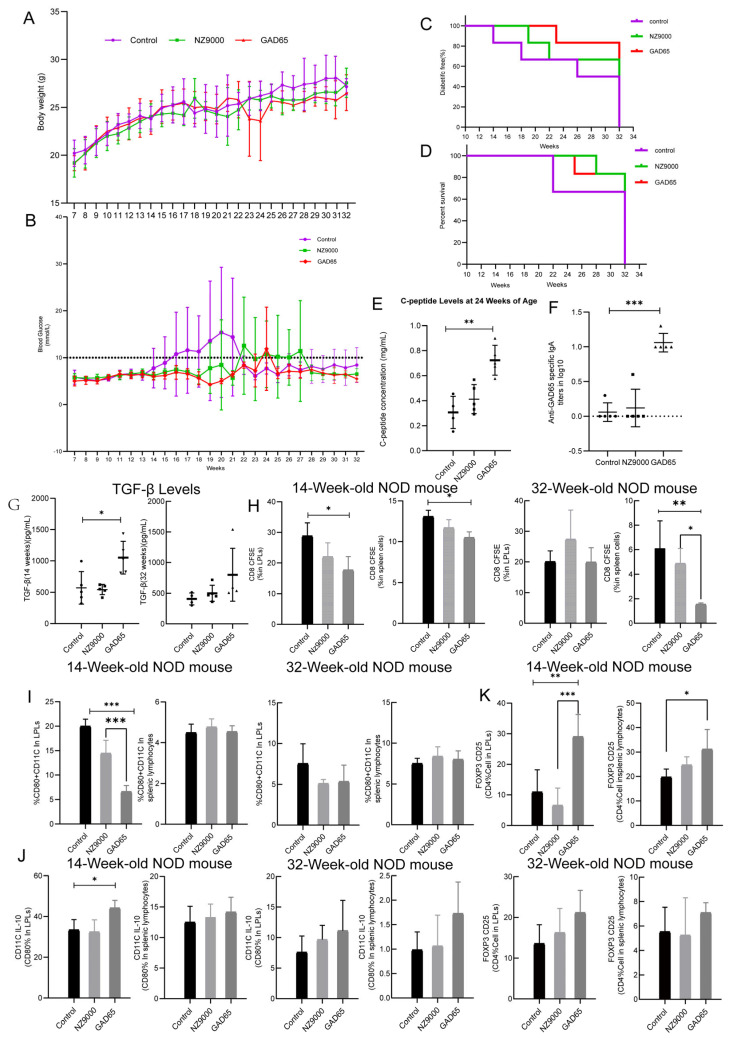
Evaluation of vaccine efficacy. (**A**,**B**) Body weight and blood glucose monitoring in NOD mice from 7 to 32 weeks of age. (**C**) Diabetes-free incidence in differently treated NOD mice. (**D**) Mortality rates across groups within 32 weeks. (**E**) C-peptide expression levels. (**F**) Anti-GAD65-specific IgA and (**G**) Serum TGF-β concentration at week 14 and 32. (**H**) GAD65 vaccine suppresses CD8^+^ T lymphocytes. Intestinal LPLs and splenic lymphocytes were collected at 14 and 32 weeks for flow cytometry analysis of CD8^+^ T cells. (**I**,**J**) GAD65 vaccine inhibits DC maturation while increasing IL-10-secreting immature DCs. (**K**) GAD65 vaccine enhances Treg cell proportions. Flow cytometry was performed on LPLs and splenic lymphocytes at 14 and 32 weeks. In the test of 14th week, n = 5. In the test of 32nd week, due to death caused by diabetes, n = 4–5.* *p* < 0.05, ** *p* < 0.01, *** *p* < 0.001. The data for groups control and GAD65 at the 14th week (**F**–**K**) are from our previous work [9], and are presented here for comparison.

As shown in Figure 1A, no significant differences in body weight were observed among the groups. However, at 23–24 weeks, one mouse in the GAD65-*L. lactis* group experienced a significant decrease in body weight. Continuous blood glucose monitoring revealed that the GAD65-*L. lactis* delayed the onset of T1D in NOD mice from 14 to 22 weeks of age (Figure 1B). At 23–24 weeks, the brief increase in blood glucose in the GAD65-*L. lactis* group was associated with a significant decrease in body weight at the same period. At 32 weeks of age, the GAD65-*L. lactis* group exhibited a protection efficacy of 83% (5/6), while the NZ9000-*L. lactis* group showed 66.6% efficacy (4/6). In contrast, the control group demonstrated a protection efficacy of 50% at 32 weeks (Figure 1C). Kaplan–Meier survival analysis further indicated that both the GAD65-*L. lactis* and NZ9000-*L. lactis* groups had significantly prolonged survival times compared to the control group (Figure 1D). As shown in Figure 1E, at 24 weeks of age, C-peptide levels in the GAD65-*L. lactis* group were significantly higher than those in the control group (*p* < 0.05), while the NZ9000-*L. lactis* group also exhibited markedly elevated levels compared to controls.

### 3.3. The Effect of GAD65-L. lactis on Humoral Immunity

At 14 weeks, the GAD65-*L. lactis* elicited GAD65-specific IgA (Figure 1F) with a titer of 1:10 in NOD mice at two weeks post-final immunization (*p* < 0.05).

### 3.4. The Effect of GAD65-L. lactis on Cellular Immunity

At 14 weeks of age, serum TGF-β concentration increased significantly in the GAD65-*L. lactis* group (Figure 1G, *p* < 0.05). By 32 weeks of age, although the serum TGF-β concentration did not show a significant increase compared to the control group (Figure 1G, *p* > 0.05), an upward trend was observed in both the GAD65-*L. lactis* group and the NZ9000-*L. lactis* group. Notably, the increase was particularly pronounced in the GAD65-*L. lactis* group.

We measured the proportions of CD8^+^ T cells, CD80^+^ CD11c^+^ cells, IL-10-secreting CD11c^+^ DCs, and Treg cells in NOD mice at 14 and 32 weeks of age. As shown in Figure 1H, at 14 weeks, the GAD65-*L. lactis* significantly suppressed CD8^+^ T cells in both intestinal LPLs and splenic lymphocytes (*p* < 0.05) compared with the control group. This suppressive effect in the intestinal LPLs weakened by 32 weeks, whereas it still existed and exerted a significant effect in the splenic lymphocytes compared with the control group (*p* < 0.001) and NZ9000-*L. lactis* group (*p* < 0.05). At 14 weeks, the GAD65-*L. lactis* group significantly inhibited GAD65-specific mature DC populations within the intestinal LPLs compared with the control group and NZ9000-*L. lactis* group (Figure 1I, *p* < 0.001). This inhibitory effect diminished over time, and no significant difference was observed by week 32 (*p* > 0.05). However, within splenic lymphocytes, no significant differences were observed at either 14 or 32 weeks (*p* > 0.05); nevertheless, by week 32, all groups showed a decrease in immature DC cell numbers. More intriguingly, Figure 1J shows that at 14 weeks, the proportion of IL-10-secreting immature DCs within LPLs was significantly increased in the GAD65-*L. lactis* group (*p* < 0.05). By week 32, however, although the proportion of IL-10-secreting immature DCs in the GAD65-*L. lactis* group showed an increase compared to the control group, this difference was not statistically significant. No significant intergroup differences were observed in splenic lymphocytes at either time point. Furthermore, a general downward trend in IL-10-secreting DCs was evident from 14 to 32 weeks. Compared to the control group and the NZ9000-*L. lactis* group at 14 weeks, the GAD65-*L. lactis* significantly induced the expansion of Treg cells in both intestinal and splenic tissues (Figure 1K, *p* < 0.05). Notably, the induction of Treg cells in LPLs was even more pronounced, with *p*-values less than 0.01 and 0.001, respectively. Over time, although the proportion of Tregs in the GAD65-*L. lactis* group remained higher than that in the control group and NZ9000-*L. lactis* group, this difference became statistically non-significant, indicating that their inductive effect gradually diminished.

### 3.5. Alterations in Gut Microbial Diversity Following Oral Administration of L. lactis-Based Vaccine

**Figure 2:**
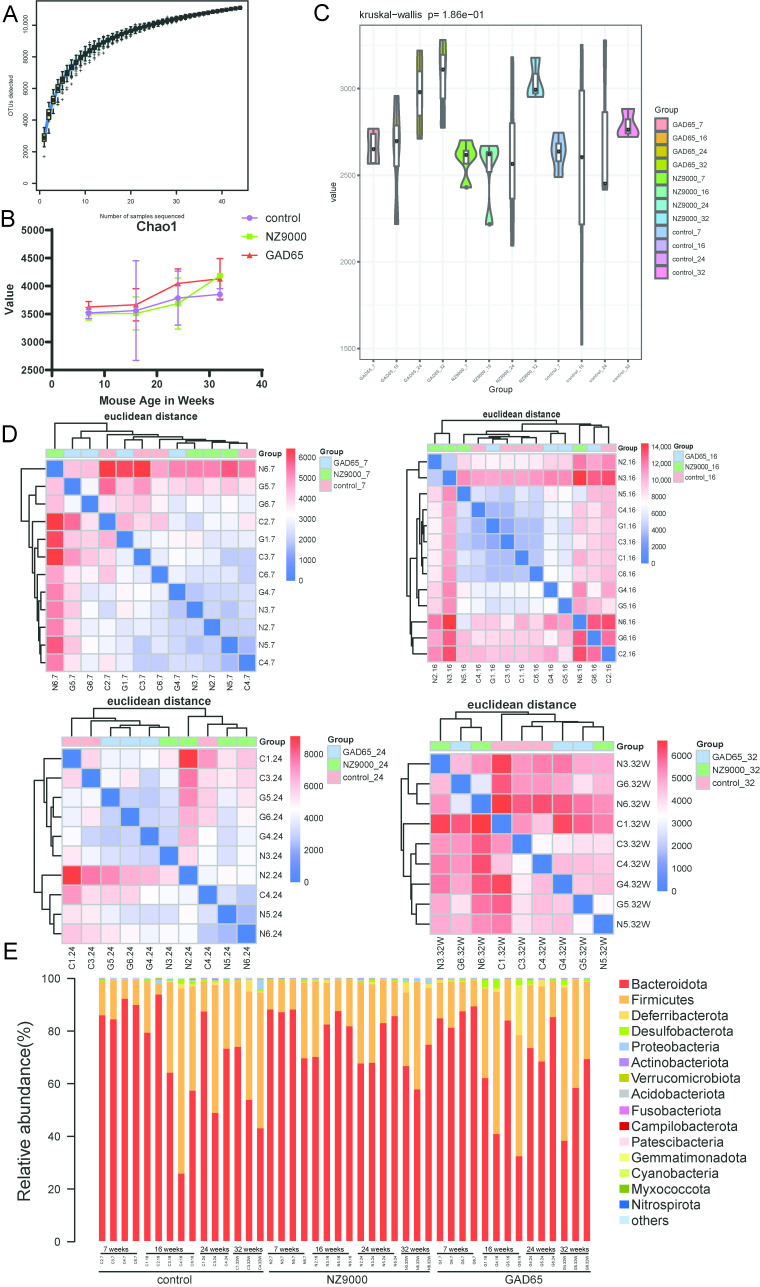
Integrated Profiling of Gut Microbial Composition and Diversity. (**A**) Species accumulation curves, with the *x*-axis representing the sample size and the *y*-axis representing the number of observed OTUs. (**B**,**C**) Distribution of alpha diversity indices across groups and significance of intergroup differences. In B, the *x*-axis indicates mouse age in weeks, and the *y*-axis shows the index values. In C, the *x*-axis represents mouse age in weeks, and the *y*-axis represents the alpha diversity index. (**D**) Heatmap of sample distances, where cooler colors (blue) indicate closer sample distances and higher similarity, while warmer colors (red) indicate greater distances. (**E**) Bar plot of the Top 15 community composition of phylum level, where each bar represents a sample, colors denote different taxonomic annotations, and “Others” represents all species beyond the Top 15. For each time point and research group, a minimum of three samples is required, ensuring at least three replicates.

High-throughput sequencing of the 16S rRNA gene was performed on all samples, yielding high-quality valid sequences after quality control and denoising. These sequences were clustered into 11,096 operational taxonomic units (OTUs) based on a 97% similarity threshold. The rarefaction curves for all samples plateaued (Figure 2A), indicating that the current sequencing depth was adequate to capture the majority of microbial species present in the samples.

To evaluate the microbial community diversity within samples from each group, we calculated the Alpha diversity indices (Supplementary Table S1). As shown in Figure 2B,C, the species richness index and Chao1 index were significantly increased in the group orally administered the GAD65-*L. lactis* vaccine compared to the control group. These results indicate that oral administration of the GAD65-*L. lactis* vaccine significantly enhances both the species richness and overall diversity of the microbial community.

Analysis based on Euclidean distance (Figure 2D) revealed that at 7 weeks of age, NOD mice exhibited close inter-sample distances, high similarity, and minimal baseline differences among groups. Over time, the plot displayed progressively redder coloration, indicating that both GAD65-*L. lactis* and NZ9000 *L. lactis* vaccine interventions altered the overall community structure of gut microbiota and increased its diversity. By 32 weeks, substantial distances were observed both between and within groups, accompanied by markedly low similarity.

**Figure 3:**
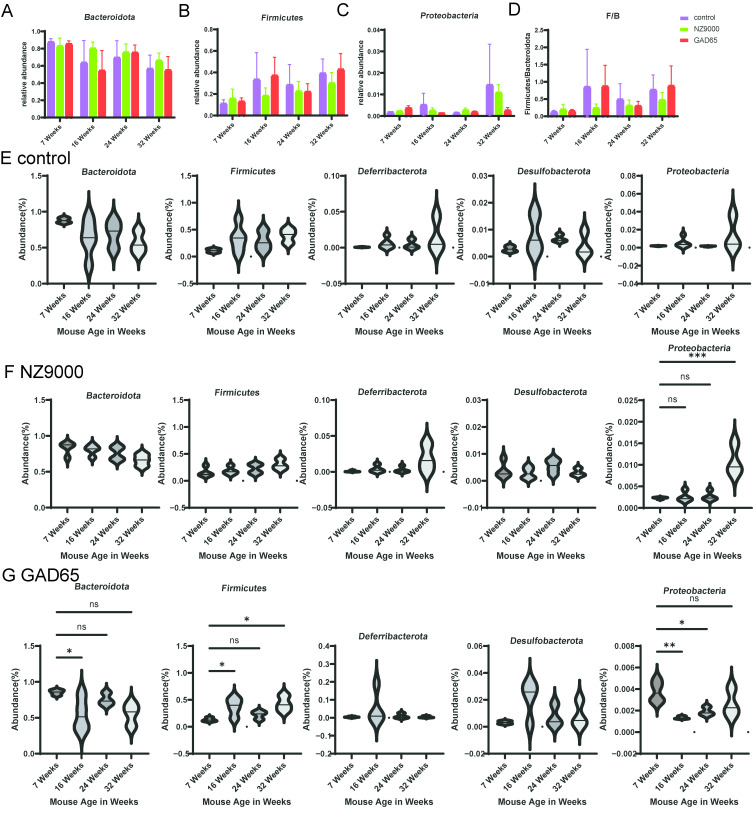
Alterations in Bacterial Abundance at the Phylum Level. (**A**–**C**) Comparison of the relative abundance of *Bacteroidota*, *Firmicutes*, and *Proteobacteria* among the three groups across different time points. (**D**) Temporal changes in the F/B ratio. (**E**–**G**) Time-series plots of gut microbiota relative abundance changes at the phylum level within the same treatment groups across different time points. The longitudinal analysis of the top five most abundant phyla across the three groups is presented. The Wilcoxon rank sum test was used to analyze statistical significance between different time points, with asterisks indicating * *p* < 0.05, ** *p* < 0.01 and *** *p* < 0.001. ns denotes no significant difference.

As illustrated in Figure 2E, the top five bacterial phyla identified are *Bacteroidota*, *Firmicutes*, *Deferribacterota*, *Desulfobacterota*, and *Proteobacteria*. Among these, *Bacteroidota* and *Firmicutes* predominantly constitute the gut microbiota. These two phyla accounted for approximately 95% of sequence reads at every time point. In the control group, the proportion of *Bacteroidota* began to decrease starting at 16 weeks, and this decline became more pronounced over time (Figure 3A). In contrast, supplementation with either GAD65-*L. lactis* or NZ9000 *L. lactis* effectively reversed this declining trend. Following the administration of the GAD65-*L. lactis* vaccine, a progressive increase in the abundance of the *Firmicutes* phylum was observed over time (Figure 3B). Additionally, our study identified an elevated *Firmicutes* to *Bacteroidota* (F/B) ratio (Figure 3D) in both the NZ9000-*L. lactis* and GAD65-*L. lactis* groups, accompanied by a relative decline in the *Proteobacteria* phylum (Figure 3C).

**Figure 4:**
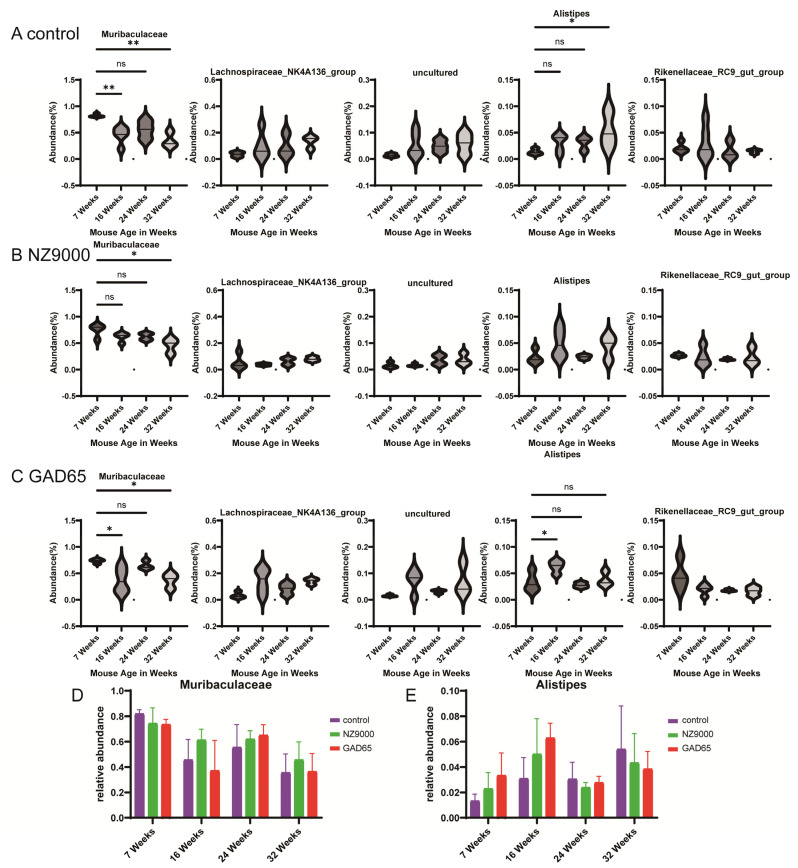
Alterations in Bacterial Abundance at the Genus Level. (**A**–**C**) Time-series plots of gut microbiota relative abundance changes at the Genus level within the same treatment groups across different time points. The longitudinal analysis of the top five most abundant genera across the three groups is presented. The Wilcoxon rank sum test was used to analyze statistical significance between different time points, with asterisks indicating * *p* < 0.05 and ** *p* < 0.01. ns denotes no significant difference. (**D**,**E**) Comparison of the relative abundance of *Muribaculaceae* and *Alistipes* among the three groups across different time points.

The Wilcoxon rank sum test was employed to examine the top five most abundant phyla (Figure 3E–G, Supplementary Table S2) and genera (Figure 4A–C, Supplementary Table S3), with the aim of elucidating the effects of the GAD65-*L. lactis* and NZ9000 *L. lactis* on the intestinal microbiota. As demonstrated in Figure 3G, the abundance of the *Bacteroidota* phylum in the GAD65-*L. lactis* group at 16 weeks of age was significantly reduced (*p* < 0.05) compared to its abundance at 7 weeks of age. In contrast, the abundance of the *Firmicutes* phylum in the GAD65-*L. lactis* group was significantly increased (*p* < 0.05) at both 16 and 32 weeks of age relative to 7 weeks of age. Consequently, the *Firmicutes*/*Bacteroidota* (F/B) ratio (Figure 3D) at these time points was substantially higher in the GAD65-*L. lactis* group than in the other two groups. Additionally, the abundance of the *Proteobacteria* phylum in the GAD65-*L. lactis* group was significantly decreased at 16 (*p* < 0.01) and 24 (*p* < 0.05) weeks of age compared to 7 weeks of age. Conversely, in the NZ9000-*L. lactis* group (Figure 3F), the abundance of the *Proteobacteria* phylum was significantly elevated (*p* < 0.001) at 32 weeks of age.

At the genus level (Figure 4, Supplementary Table S3), the five most prevalent genera identified were *Muribaculaceae*, *Lachnospiraceae_NK4A136_group*, uncultured, *Alistipes*, and *Rikenellaceae_RC9_gut_group*. As the mice aged, a notable decline in the relative abundance of *Muribaculaceae* was observed across all experimental groups (Figure 4D). This decrease was especially significant in the control group at 32 weeks (Figure 4A, *p* < 0.01). In contrast, the groups that received oral vaccination with GAD65-*L. lactis* and NZ9000-*L. lactis* exhibited a more moderate change in *Muribaculaceae* abundance. Additionally, with increasing age, the relative abundance of *Alistipes* showed an upward trend across all mouse groups (Figure 4E). This increase was particularly marked in the control group at 32 weeks (Figure 4A, *p* < 0.05). The group administered with the oral GAD65-*L. lactis* vaccine displayed a significant increase in *Alistipes* abundance at 16 weeks of age (Figure 4C, *p* < 0.05). However, in these vaccinated groups, the relative abundance of *Alistipes* changed more gradually with age, a trend that was especially pronounced in the GAD65-*L. lactis* group.

### 3.6. Screening of Differential Metabolites via Untargeted Metabolomics

Untargeted metabolomic analysis of all samples was performed using the UPLC-MS/MS platform. To ensure data quality, quality control (QC) samples, prepared by pooling equal aliquots from all experimental samples, were periodically inserted into the analytical sequence. The base peak ion chromatograms (BPCs) of the QC samples showed a high degree of overlap (Supplementary Figure S2), indicating stable instrument performance and good reproducibility of chromatographic separation throughout the analytical process. Additionally, the total ion chromatograms of the 21 samples (Supplementary Figure S3) demonstrate that the baseline is generally stable with minimal noise.

**Figure 5:**
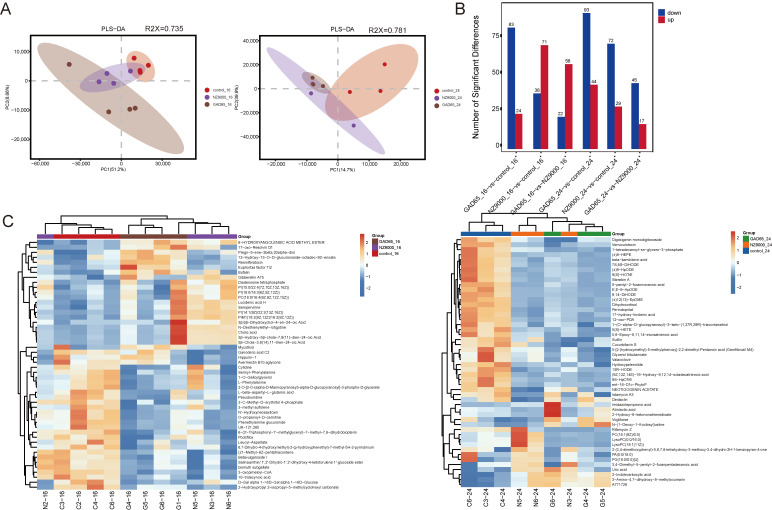
Distinct Metabolomic Signatures and Differential Metabolites. (**A**) PLS-DA score plot. The *x*-axis (PC1) and *y*-axis (PC2) represent the first and second principal components, respectively, with the numbers in parentheses indicating the corresponding percentage of variance explained. Each point in the plot represents an individual sample (at 16 weeks of age, n = 4, at 24 weeks of age, n = 3) with different colors denoting distinct sample groups. The elliptical regions represent the 95% confidence intervals, illustrating the central tendency of sample distribution. (**B**) Fold change bars. The *x*-axis represents the comparison groups, the *y*-axis shows the number of differential metabolites, with red indicating upregulated and blue indicating downregulated. (**C**) The *x*-axis denotes the sample names, while the *y*-axis represents the differential metabolites. The color gradient from blue to red indicates the expression abundance of metabolites from low to high, with red reflecting higher expression levels of differential metabolites.

To evaluate the overall variations in metabolic profiles across the groups, Partial Least Squares-Discriminant Analysis (PLS-DA) was conducted. The analysis revealed a distinct separation trend among the three sample groups at both 16 and 24 weeks (refer to Figure 5A and Supplementary Table S4). Interventions using either the GAD65-*L. lactis* vaccine or NZ9000-*L. lactis* significantly modified the metabolic state of NOD mice. The metabolomic analysis identified a total of 35,816 metabolites, of which 3853 were classified as differential metabolites (see Supplementary Table S5). The differential metabolites, identified based on the screening criteria of a *p*-value < 0.05 and a VIP score > 1.0, are depicted in Figure 5B. At 16 weeks of age, a comparative analysis of the GAD65-*L. lactis* vaccine group and the Control group identified 83 downregulated and 24 upregulated metabolites. In contrast, the NZ9000-*L. lactis* group, when compared to the Control, demonstrated 38 downregulated and 71 upregulated metabolites. A direct comparison between the two intervention groups revealed 22 downregulated and 58 upregulated metabolites. By the 24-week age, the GAD65-*L. lactis* vaccine group showed 93 downregulated and 44 upregulated metabolites relative to the Control group, whereas the NZ9000-*L. lactis* group exhibited 72 downregulated and 29 upregulated metabolites. The comparison between the two intervention groups at this time point indicated 45 downregulated and 17 upregulated metabolites. Overall, at 24 weeks of age the most pronounced metabolic differences were observed between the Control and the GAD65-*L. lactis* vaccine groups, while the least differences were noted between the NZ9000-*L. lactis* and GAD65-*L. lactis* vaccine groups, as evidenced by the respective numbers of differential metabolites.

Based on the similarity of metabolite expression patterns (using Z-score normalized expression data), bidirectional clustering analysis of both metabolites and samples was performed using a hierarchical clustering algorithm. To more intuitively display the similarities between samples, the consistency of metabolites within groups, and the differences between groups, the expression levels of all significant differential metabolites and the Top 50 most significant differential metabolites (based on VIP scores) were separately clustered and visualized (Figure 5C). The results showed distinct color patterns among the three groups, indicating metabolic differences attributable to the different treatments.

A combination of multidimensional and unidimensional analysis methods was employed to screen for differential metabolites between groups. Using the criteria of *p*-value < 0.05 and VIP > 1.0, differential metabolites were identified. These were then ranked in descending order of VIP scores for the top five both up-regulated and down-regulated metabolites, as shown in Table 2. Among them, 5-Hydroxypentanoic acid (a short-chain fatty acid) was up-regulated in the GAD65-*L. lactis* vaccine group at 16 weeks compared to the control group. The extracted chromatogram of this compound is provided in Supplementary Figure S3.

**Figure 6:**
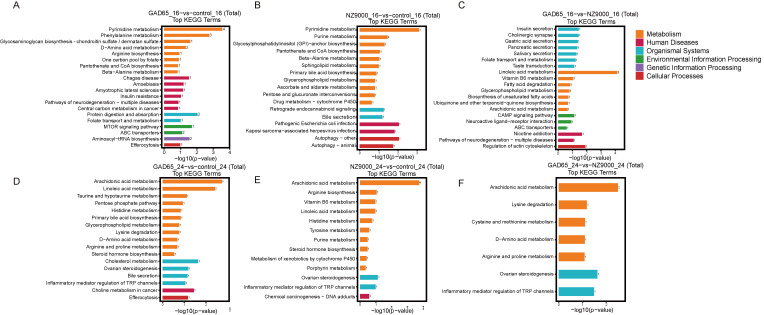
Bar Chart of Significantly Enriched KEGG Pathways (*p* < 0.05). Enriched pathways across groups at 16 and 24 weeks of age. (**A**) GAD65 vs. Control at 16 Weeks. (**B**) NZ9000 vs. Control at 16 Weeks. (**C**) GAD65 vs. NZ9000 at 16 Weeks. (**D**) GAD65 vs. Control at 24 Weeks. (**E**) NZ9000 vs. Control at 24 Weeks. (**F**) GAD65 vs. NZ9000 at 24 Weeks. The *x*-axis represents the −log10 *p*-value for each pathway, and the *y*-axis shows the names of different pathways. The numbers on the bars indicate the count of differential metabolites annotated to the respective pathway, while the varying colors of the bars represent different KEGG pathway categories.

To gain deeper insights into the biological functions of the differential metabolites, we performed KEGG pathway enrichment analysis (Figure 6). According to the KEGG analysis (Figure 6, Supplementary Table S6), at 16 weeks of age, the metabolic pathways primarily enriched in the GAD65-*L. lactis* group were pyrimidine metabolism and linoleic acid metabolism. Compared to the control group, the pyrimidine metabolism pathway was significantly downregulated in both the GAD65-*L. lactis* group and the NZ9000-*L. lactis* group (see Figure S4, Supplementary Table S7). This is consistent with the purpose of our oral GAD65-*L. lactis* administration, which is to induce immune tolerance. A state of tolerance is accompanied by metabolic quiescence, and thus, pyrimidine metabolism is naturally downregulated [21]. Additionally, a comparison between the GAD65-*L. lactis* group and the NZ9000-*L. lactis* group revealed that the linoleic acid metabolism pathway was significantly more upregulated in the GAD65-*L. lactis* group. At 24 weeks of age, compared to the control group (see Figure S4, Supplementary Table S7), the arachidonic acid metabolism pathway was significantly downregulated in both the NZ9000-*L. lactis* group and the GAD65-*L. lactis* group. When comparing the NZ9000-*L. lactis* group with the GAD65 -*L. lactis* vaccine group, the downregulation of the arachidonic acid metabolism pathway was more pronounced in the GAD65-*L. lactis* vaccine group. This indicates that both the NZ9000-*L. lactis* group and the GAD65-*L. lactis* vaccine group can downregulate the arachidonic acid metabolism pathway, with the GAD65-*L. lactis* group exhibiting a stronger suppressive effect. Compared with the control group, the NZ9000-*L. lactis* group showed that the metabolism of xenobiotics by cytochrome P450 was enriched and upregulated at both 16 weeks and 24 weeks of age.

### 3.7. Integrated Microbiome–Metabolome Analysis

**Figure 7:**
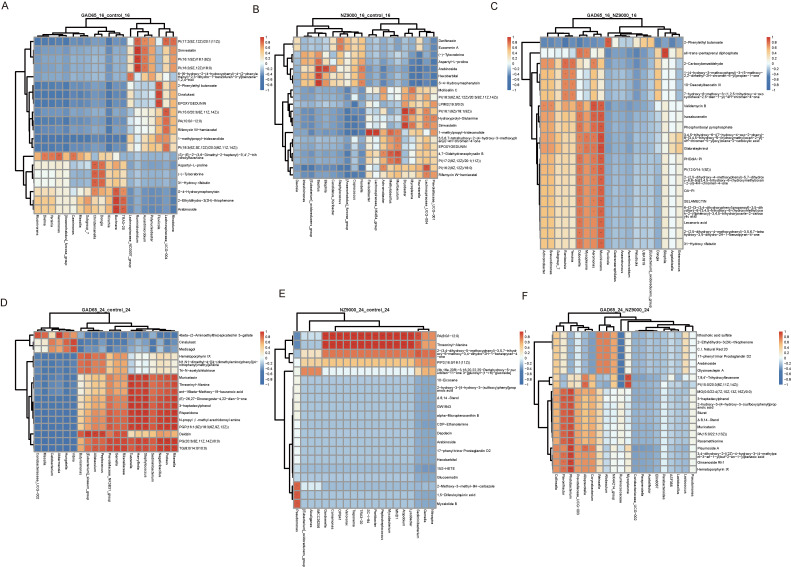
Integrated correlation heatmap visualizing associations between differential microbes and differential metabolites at the genus level across experimental groups and timepoints. This analysis was performed to elucidate the potential functional interplay between differentially abundant gut microbiota and altered metabolites following intervention with recombinant *L. lactis* strains. (**A**) GAD65 vs. Control at 16 Weeks. (**B**) NZ9000 vs. Control at 16 Weeks. (**C**) GAD65 vs. NZ9000 at 16 Weeks. (**D**) GAD65 vs. Control at 24 Weeks. (**E**) NZ9000 vs. Control at 24 Weeks. (**F**) GAD65 vs. NZ9000 at 24 Weeks. The *x*-axis represents the top 20 differentially abundant microbial genera, and the *y*-axis represents the top 20 differential metabolites. Spearman correlation analysis revealed distinct, time-dependent association patterns between specific microbial genera and host metabolites in the GAD65-*L. lactis* and NZ9000-*L. lactis* groups compared to the control. In the heatmap, “*” indicates correlations with *p* < 0.05, “**” indicates *p* < 0.01, and “***” indicates *p* < 0.001. The visualization of these specific correlations is crucial, as it moves beyond independent omics lists to propose testable mechanistic hypotheses. It directly links changes in microbial composition to alterations in the host metabolic landscape, suggesting candidate microbes and metabolites that may mediate the observed physiological effects.

Alterations in metabolic pathways may be influenced, at least partially, by the gut microbiota. To further explore the intrinsic correlations between differentially expressed metabolites and microbial communities, we performed Spearman correlation analysis to elucidate the relationships between the microbiome and metabolome. For the control group, recombinant GAD65-*L. lactis* group, and NZ9000-*L. lactis* group, we analyzed correlations between altered metabolites and differentially abundant gut microbiota at the genus level. At 16 weeks (Figure 7, Supplementary Table S8), compared to the control group, the GAD65-*L. lactis* group showed a positive correlation between *Ruminiclostridium* and various phosphatidylinositols (PIs) and simvastatin. *Ruminiclostridium* is a genus of bacteria belonging to the *Firmicutes* phylum. At 24 weeks, *Rikenella* showed a positive correlation with 3-heptadecylphenol, risperidone, and PGP(16:1(9Z)/18:3(6Z,9Z,12Z)). In the NZ9000-*L. lactis* groups, whether at 16 or 24 weeks, the patterns in the correlation heatmaps underwent global changes compared to their respective control groups. At 16 weeks, *Lachnospiraceae_UCG−004* showed a positive correlation with PI(16:2(9Z,12Z)/18:0). At 24 weeks, multiple bacterial genera showed positive correlations with PA(8:0/i-12:0) and Threoninyl-Alanine.

## 4. Discussion

Numerous studies have demonstrated that inducing immune tolerance via the GAD65 antigen can effectively prevent the onset and progression of type 1 diabetes (T1D) [22,23,24]. By integrating animal experiments, 16S rRNA sequencing, and untargeted metabolomics, this research elucidates the comprehensive protective effects of an orally administered GAD65-*L. lactis* delivered by NZ9000 *L. lactis* in T1D intervention.

In animal models, mice treated with oral GAD65-*L. lactis* or NZ9000-*L. lactis* exhibited increased survival rates and delayed disease onset. Additionally, the C-peptide secretion in mice from the GAD65-*L. lactis* group was elevated, suggesting partial preservation of islet function. Following oral administration of GAD65-*L. lactis*, analysis of LPLs in the intestinal tract demonstrated a significant reduction in the number of GAD65-specific CD8^+^ T cells and mature DCs. Conversely, there was an increase in the number of GAD65-specific immature DCs secreting IL-10, alongside an elevated proportion of Tregs and enhanced secretion of TGF-β. These findings suggest that the GAD65-*L. lactis* vaccine effectively induced GAD65-specific immune tolerance within the mouse intestinal tract. Although NZ9000-*L. lactis* did not elicit GAD65-specific immune tolerance, NOD mice treated solely with NZ9000-*L. lactis* exhibited less severe disease symptoms compared to the control group. This protective effect can likely be attributed to the inherent probiotic properties of *L. lactis.* These properties promoted early but transient immunomodulatory changes, such as the temporary inhibition of DC maturation and the induction of Tregs. These changes were mainly localized within the gut compartment [25].

Furthermore, post-vaccination analysis revealed that *Bacteroidota* and *Firmicutes* emerged as the predominant phyla, while *Muribaculaceae* and *Alistipes* became the dominant genera within the intestinal microbiota. At 16 weeks of age, both the GAD65-*L. lactis* and NZ9000-*L. lactis* groups exhibited a significant enrichment in pyrimidine metabolism compared to the control group. However, when comparing the GAD65-*L. lactis* group to the NZ9000-*L. lactis* group, there was a more pronounced enrichment in linoleic acid metabolism. By 24 weeks of age, the arachidonic acid metabolism pathway showed the highest level of enrichment across all group comparisons. Additionally, the NZ9000-*L. lactis* group, relative to the control group, demonstrated an enrichment and upregulation in the metabolism of xenobiotics by cytochrome P450 at both 16 and 24 weeks of age.

Integrated Microbiome–Metabolome Analysis revealed that in the GAD65-*L. lactis* groups, *Ruminiclostridium* exhibited a positive correlation with various PIs and simvastatin at the 16 week, whereas *Rikenella* was correlated with 3-heptadecylphenol, risperidone, and PGP(16:1(9Z)/18:3(6Z,9Z,12Z)) at 24 weeks. In contrast, the NZ9000-L. lactis groups demonstrated widespread changes in correlation patterns. Notably, *Lachnospiraceae_UCG−004* was positively correlated with PI(16:2(9Z,12Z)/18:0) at 16 weeks, and several genera were correlated with PA(8:0/i-12:0) and Threoninyl-Alanine at 24 weeks.

Our study revealed the dynamic impact of the GAD65-*L. lactis* on the gut microbiota of NOD mice through 16S rRNA sequencing. At the phylum level, the proportion of *Firmicutes* increased in both the NZ9000-*L. lactis* and GAD65-*L. lactis* groups. *Firmicutes* are a major bacterial phylum colonizing the healthy human gut. Recent studies have discovered that *Firmicutes* possess many genes responsible for fermenting dietary fiber, producing SCFAs, and interacting with the intestinal mucosa, thereby contributing to homeostasis [26]. These SCFAs have significant effects on immune cells and the gut, providing an energy supply and potentially extending the lifespan of diabetic mice [27]. Studies have established a link between gut microbiota dysbiosis and the pathogenesis of Type 1 Diabetes (T1D) in susceptible individuals. This dysbiosis, characterized by a depletion of short-chain fatty acid (SCFA)-producing bacteria, may trigger immune responses that promote disease onset [2,3]. The significance of SCFAs is further highlighted by their role in promoting the differentiation of regulatory Tregs [28], which may explain the observed increase in Treg proportions within gut and splenic lymphocytes two weeks post-immunization in our study.

Our findings revealed specific shifts in the gut microbiota composition, including an elevated *Firmicutes*/*Bacteroidota* (F/B) ratio and a relative reduction in *Proteobacteria* in the treatment groups. An increase in *Bacteroidota* has been identified as a potential biomarker for T1D onset [29,30]; therefore, the elevated F/B ratio suggests a beneficial modulation. Concurrently, the decrease in *Proteobacteria* is a positive indicator, as this phylum contains numerous opportunistic pathogens. A reduction in its abundance typically signifies a more stable and healthier gut environment, a principle underscored by its importance in managing conditions like bronchiectasis [31].

Moreover, the interaction between gut microbiota and host immunity is influenced by bacterial metabolites. Various bacterial phyla, including *Actinobacteria*, *Firmicutes*, *Bacteroidota*, *Proteobacteria*, and *Fusobacteria*, metabolize tryptophan (Trp) into metabolites such as kynurenine (Kyn) [32]. These metabolites activate the aryl hydrocarbon receptor (AhR) with differing affinities [33], which subsequently modulates the activity of indoleamine 2,3-dioxygenase 1 (IDO1). The AhR-IDO1 axis constitutes a crucial pathway for regulating the host’s Tregs, thereby impacting immune homeostasis and disease susceptibility.

At the genus level, the *Muribaculaceae* family was identified as the predominant bacterial group. Members of the *Muribaculaceae* family are known to produce SCFAs from both endogenous and exogenous polysaccharides [34]. Specifically, these bacteria can synthesize propionate, and the production of SCFAs such as propionate and butyrate has been associated with increased lifespan in diabetic mice [35]. In counteracting the observed decline in *Muribaculaceae* over time within the control group, NZ9000-*L. lactis* demonstrated a more rapid effect compared to GAD65-*L. lactis*. This observation suggests that NZ9000-*L. lactis* functions as an initial force in establishing a dominant gut microbiota, whereas GAD65-*L. lactis* contributes to the later-stage consolidation and enhancement of specific immune responses.

*Lactobacilli* serve as both effective probiotics and oral vaccine vectors. As probiotics, they modulate gut microbiota by influencing bacteria like *Alistipes* and engaging in cross-feeding with *Muribaculaceae*, thereby promoting gut health [36]. As delivery vectors, they encapsulate antigens to protect against degradation, enhancing antigen stability and vaccine efficacy. These functions, combined with their inherent ability to promote Treg induction [37], make *Lactobacilli* a versatile platform for positively shaping host immunity.

A recent study demonstrated that oral administration of *Alistipes* can prolong the survival of mildly mismatched skin grafts in mice, suggesting its role in inducing host immune tolerance [38]. In our study, fluctuations in the abundance of *Alistipes* were observed in the GAD65-L. lactis vaccine group at 16 weeks of age. Corroborating this potential shift, we measured a significant increase in TGF-β levels in the same group, confirming the establishment of a local tolerogenic environment.

These findings collectively highlight the synergistic interactions among the GAD65 antigen, the *Lactococcus lactis* vector, and the gut microbiota in metabolic and immune regulation. Given that progressive severe diabetes and malnutrition during the advanced disease stage led to a concurrent decline in therapeutic effects across all three groups, and substantial intra-group variability made it difficult to distinguish between the effects of the vaccine and the empty vector intervention, this study selected 16 and 24 weeks as critical timepoints for metabolomics analysis.

Metabolomics acts as a clear mirror, reflecting the subtle yet authentic physiological responses of an organism to genetic or environmental changes. Despite the differences in species composition between the gut microbiota of NOD mice and humans, a growing body of research indicates that the microbiome’s influence on the host is primarily mediated through a series of key small-molecule metabolites produced or transformed by these microbes, which are highly conserved across mammalian species. Large-scale metagenomic studies have demonstrated that, although the microbiota of healthy individuals exhibit species-level diversity, they maintain a high degree of consistency in core metabolic pathways [39]. Notably, experimental evidence has shown that when human microbiota is transplanted into mice, the characteristic metabolic profiles can be accurately reconstructed [40]. This study aims to utilize the NOD mouse model to investigate the specific impact of GAD65-L. lactis on the host metabolic network.

A crucial and intriguing finding is that NZ9000-*L. lactis*, the delivery vector used in this experiment, is not merely a passive antigen carrier but exhibits proactive and significant metabolic regulatory effects—a discovery of paramount importance.

The metabolic differences between the GAD65-*L. lactis* group and the NZ9000-*L. lactis* group were far smaller than the differences between each of them and the control group. This suggests that NZ9000-*L. lactis* alone has already induced alterations in the metabolomic profile of NOD mice. We observed this earlier during the immunological evaluation of the vaccine: at 24 weeks of age, C-peptide levels increased in both the NZ9000-*L. lactis* and GAD65-*L. lactis* groups. Although the rise in the NZ9000-*L. lactis* group was less pronounced than in the oral vaccine group, it still represented a substantial increase compared to the control group. NZ9000-*L. lactis* may act as a metabolic primer that potentiates the efficacy of GAD65-*L. lactis.*

Based on the KEGG pathway enrichment analysis results, this study reveals a novel mechanism by which the oral GAD65-*L. lactis* vaccine induces immune tolerance from a metabolic perspective. At 16 weeks post-immunization, the vaccine group exhibited significant downregulation of pyrimidine metabolism, a phenomenon closely associated with the establishment of immune tolerance. Notably, as pyrimidine metabolism serves as a crucial pathway for nucleic acid synthesis during cell proliferation, its downregulation precisely corroborates the suppressive effect of vaccine-activated immunosuppressive Tregs on effector T cell proliferation. This metabolic reprogramming demonstrates that the vaccine promotes the formation of an immune-tolerant microenvironment not only through regulating immune cell subsets but also by reshaping the host’s metabolic state, providing a new metabolic dimension for understanding the immunoregulatory mechanisms of oral vaccines. In our study, the NZ9000-*L. lactis* group exhibited upregulation of xenobiotic metabolism mediated by cytochrome P450 at both 16 and 24 weeks of age. Previous research has indicated that cytochrome P450 (CYP)-dependent metabolism of exogenous substances can be indirectly regulated by the aryl hydrocarbon receptor (AHR) [41]. Additionally, cytochrome P450 1A2 (CYP1A2), a specific CYP enzyme, has been found to co-localize with areas of high infiltration by immunosuppressive Tregs [42].

Compared with the NZ9000-*L. lactis* group, a more pronounced up-regulation of the linoleic acid metabolism pathway was observed in the GAD65-*L. lactis* group. This indicates that the effect is not merely due to the vector but results from the vaccine antigen specifically activating the host’s metabolic and immune responses. Additionally, upstream products of the linoleic acid metabolism pathway can provide essential membrane components for plasma cells, preparing the body for both local (e.g., IgA) and systemic immunity [43]. In future studies, the activity level of the linoleic acid metabolism pathway could potentially serve as a biomarker to predict or evaluate the immunogenicity of oral vaccines.

At 16 weeks of age, compared to the control group, the arachidonic acid metabolism pathway was significantly downregulated in both the NZ9000 and GAD65-*L. lactis* vaccine groups. When comparing the NZ9000-*L. lactis* group to the GAD65-*L. lactis* vaccine group, the downregulation was more pronounced in the GAD65-*L. lactis* vaccine group. This suggests that the NZ9000 vector establishes an anti-inflammatory baseline, while the GAD65-*L. lactis*, through antigen-specific immunomodulation, more potently “shuts down” this key pro-inflammatory pathway—arachidonic acid metabolism [44]. This mechanism is likely one of the core ways in which the vaccine prevents or delays the autoimmune attack on pancreatic β-cells.

Both *Muribaculaceae* and *Firmicutes* can produce SCFAs. The 5-Hydroxypentanoic acid detected through metabolomics belongs to a type of SCFA. Studies have shown that SCFAs produced by the gut microbiota possess beneficial anti-inflammatory and gut homeostatic effects, and can prevent T1D. Targeting dietary SCFAs may represent a mechanism to modify immune architecture, promote immune tolerance, and improve glycemic control for treating T1D [45].

Integrated analysis of gut microbiota and metabolites revealed the intrinsic connections through which the vaccine regulates the host. In this study, correlation analysis showed that at the 16-week mark, when the vaccine exerts its key effects, a positive correlation was observed between *Ruminiclostridium* and various phosphatidylinositols in the GAD65-*L. lactis* group. This finding holds dual significance: on one hand, as a potential short-chain fatty acid-producing bacterium [46], the increased abundance of *Ruminiclostridium* may be associated with the formation of an immune-tolerant microenvironment; on the other hand, phosphatidylinositols are key lipid molecules in cell membrane signal transduction [47], and their altered levels may reflect changes in membrane homeostasis and functional status of intestinal immune cells (such as DCs and T cells) under vaccine intervention. This suggests that the vaccine may influence the lipid metabolism and signal transduction of local intestinal immune cells by modulating specific microbial populations, thereby creating favorable conditions for the induction and functional execution of Tregs. Furthermore, at the later disease stage of 24 weeks, we observed a positive correlation between *Rikenella* and metabolites such as risperidone. Although the biological significance of risperidone as an exogenous drug remains unclear, this association indicates that both the NZ9000 vector and the GAD65-*L. lactis* intervention can significantly alter the host-microbial co-metabolic network.

This perspective fundamentally changes our interpretation of the experimental results. NZ9000-*L. lactis* is not just a delivery vehicle; it functions more like a synergistic metabolic modulator. This synergistic relationship between NZ9000-*L. lactis* and GAD65-*L. lactis* provides a new dimension and conceptual framework for understanding metabolic regulation within complex biological systems.

Despite demonstrating the therapeutic potential of the GAD65-*L. lactis* and NZ9000-*L. lactis* in ameliorating diabetic phenotypes, this study has several limitations: 1. Sample size constraints (n = 14 per group) may restrict statistical power for detecting subtle intergroup differences, necessitating validation in larger cohorts. 2. Critical data gaps at week 32 emerged due to progressive severe diabetes and malnutrition, precluding 16S rRNA sequencing and metabolomic analyses at this terminal stage, thereby limiting insights into long-term microbial/metabolic dynamics. 3. One limitation of this study is the absence of a proteomic analysis to quantify the expression levels of proteins associated with inflammation. Although our metabolomic and microbiome data strongly indicate an anti-inflammatory effect, obtaining direct evidence at the protein level, such as the quantification of cytokines or signaling molecules, would offer a more mechanistic insight into the observed phenotypic improvements.

## 5. Conclusions

Our study demonstrates that the oral GAD65-*L. lactis* vaccine effectively delays T1D progression in NOD mice through synergistic immunomodulatory, microbial, and metabolic regulation. It induces GAD65-specific immune tolerance while promoting gut microbiota remodeling and time-dependent metabolic shifts—notably suppressing pro-inflammatory arachidonic acid metabolism and enhancing linoleic acid pathways. Significantly, the empty vector NZ9000 alone establishes an “anti-inflammatory foundation” by inducing similar immunometabolic improvements, which are further amplified by GAD65 antigen to achieve enhanced suppression of key inflammatory pathways. Our findings resonate with the fundamental role of Tregs in maintaining immune homeostasis. The vaccine-driven expansion of Tregs and IL-10^+^ immature dendritic cells provides experimental support for the therapeutic potential of harnessing Treg-mediated tolerance. This synergy between a probiotic vector and a beta-cell autoantigen offers a promising translational strategy for diabetes prevention, highlighting the broader applicability of Treg-centric therapies in autoimmune disease intervention.

## Figures and Tables

**Table 1:** Animal groups and dosage.

Groups	Dose	Number (NOD)
control	100 µL PBS	14
GAD65	100 µL (10^10^ CFU/mL)/10^9^ CFU/mouse	14
NZ9000	100 µL (10^10^ CFU/mL)/10^9^ CFU/mouse	14

All mice were orally immunized for 7 consecutive days, followed by another 7 consecutive days at an interval of 2 weeks, and the food was stored away 6 h before immunization. GAD65: recombinant huGAD65 *L. lactis* vaccine; NZ9000: *L. lactis* NZ9000 with pNZ8148; control: PBS.

**Table 2:** The top five up-regulated or down-regulated differential metabolites screened by VIP values in descending order.

Metabolites	VIP	*p*-Value	log2FoldChange	Regulation
**GAD65_16-vs.-control_16**				
L-Alloisoleucine	14.6176	0.00695	1.14379	Down
3,4-Dihydro-2H-1-benzopyran-2-one	12.7497	0.03683	1.23439	Down
2-Acetyl-1,5,6,7-tetrahydro-6-hydroxy-7-(hydroxymethyl)-4H-azepine-4-one	8.03661	0.00385	0.94877	Down
2-C-Methyl-D-erythritol 4-phosphate	6.65553	0.02902	1.22807	Down
THTC	6.15843	0.00932	1.39183	Down
Dimetacrine tartrate	5.68664	0.04027	−1.2281	Up
6-Hydroxyangolensic Acid Methyl Ester	2.2016	0.01755	−0.7035	Up
PC(DiMe(11,5)/MonoMe(11,3))	2.00352	0.0334	−2.1317	Up
Euphorbia factor Ti_2_	1.59097	0.03643	−1.7298	Up
5-Hydroxypentanoic acid	1.56521	0.01675	−0.7437	Up
**NZ9000_16-vs.-control_16**				
Phenethylamine glucuronide	4.48271	0.00479	1.89505	Down
MG(0:0/16:1(9Z)/0:0)	4.37551	0.02216	1.1632	Down
Deoxyinosine	4.10991	0.0031	1.26439	Down
Telbivudine	3.98936	0.03282	0.72069	Down
Ganoderic acid C_2_	3.76156	0.00152	1.50722	Down
(22R)-3alpha,7alpha,22-Trihydroxy-5beta-cholan-24-oic Acid	57.3482	0.0488	−1.5948	Up
3a,4b,12a-Trihydroxy-5b-cholanoic acid	42.7362	0.03138	−3.3462	Up
3α-Hydroxy-5β-chola-7,9(11)-dien-24-oic Acid	25.5582	0.02002	−2.4503	Up
5β-Chola-3,8(14),11-trien-24-oic Acid	19.5152	0.02147	−2.9188	Up
PI(16:0/18:0)	19.1612	0.04699	−5.4381	Up
**GAD65_16-vs.-NZ9000_16**				
N-Acetyl-D-glucosamine	8.9589	0.0309	0.65822	Down
Desglymidodrine	3.777	0.04995	0.53344	Down
Acetylcholine	3.43218	0.04823	0.42249	Down
Acipimox (5-methylpyrazine-2-carboxylic acid)	2.96428	0.00059	0.59656	Down
6-({3,5-dihydroxy-2-[hydroxy({2,3,4-trihydroxy-6-oxo-3-[3,4,5-trihydroxy-6-(hydroxymethyl)oxan-2-yl]cyclohexa-1,4-dien-1-yl})methyl]oxan-4-yl}oxy)-3,4,5-trihydroxyoxane-2-carboxylic acid	2.47561	0.02144	1.7766	Down
xi-2,3-Dihydro-2-oxo-1H-indole-3-acetic acid	9.93164	0.03713	−0.8193	Up
N1,N8-Diacetylspermidine	9.02402	0.01602	−1.0486	Up
Hippurin-1	7.81028	0.04452	−1.1046	Up
M 344	5.64693	0.02168	−1.4341	Up
(9Z,12Z,14E)-16-Hydroxy-9,12,14-octadecatrienoic acid	5.63666	0.04472	−0.4385	Up
**GAD65_24-vs.-control_24**				
Taurocholic acid	11.8384	0.03989	2.19745	Down
(9Z,12Z,14E)-16-Hydroxy-9,12,14-octadecatrienoic acid	8.46122	0.02868	1.77733	Down
(±)12(13)-EpOME	8.19732	0.00271	2.06938	Down
E,E-9-HpODE	6.12916	0.01663	2.53013	Down
LysoPC(0:0/16:0)	5.8749	0.02692	1.35126	Down
3-Amino-4,7-dihydroxy-8-methylcoumarin	15.4917	0.04592	-1.4871	Up
5-Aminopentanoic acid	9.89937	0.01249	-0.6199	Up
Gaboxadol	3.78973	0.004	-0.4838	Up
(3R)-3,4-Dihydroxy-3-(hydroxymethyl)butanenitrile 4-glucoside	3.45038	0.03324	-1.0679	Up
2-(2,4-dihydroxy-5-methoxyphenyl)-3-(3,7-dimethylocta-2,6-dien-1-yl)-5-hydroxy-8,8-dimethyl-4H,8H-pyrano[3,2-g]chromen-4-one	2.12585	0.01757	-1.4876	Up
**NZ9000-vs.-control_24**				
(9Z,12Z,14E)-16-Hydroxy-9,12,14-octadecatrienoic acid	9.65671	0.0021	1.92272	Down
(±)12(13)-EpOME	9.1705	0.00855	2.11728	Down
E,E-9-HpODE	6.76751	0.01541	2.40964	Down
Atrolactic acid	6.7476	0.00804	0.96461	Down
17-hydroxy-linolenic acid	6.48908	0.02538	1.87306	Down
7-Ketodeoxycholic acid	9.82503	0.01961	−0.6443	Up
Uric acid	3.27161	0.01999	−2.4394	Up
17R-HDHA	3.1493	0.0434	−0.8553	Up
Asymmetric dimethylarginine	2.71701	0.04101	−0.8598	Up
Allopregnanalone sulfate	2.60127	0.00377	−2.6514	Up
**GAD65_24-vs.-NZ9000_24**				
17-HOME(9Z)	5.92395	0.02213	0.83256	Down
(23S,24S)-17,23-Epoxy-24,29-dihydroxy-27-norlanost-8-ene-3,15-dione	5.85579	0.048	0.74105	Down
2-(3,4-dimethoxyphenyl)-5,6,7,8-tetrahydroxy-3-methoxy-3,4-dihydro-2H-1-benzopyran-4-one	5.43801	0.01118	0.66076	Down
Glycerol tributanoate	5.34647	0.00572	1.67588	Down
3,4-Dimethyl-5-pentyl-2-furanpentadecanoic acid	4.97022	0.04797	1.26745	Down
5-Aminopentanoic acid	10.9735	0.01238	−0.5518	Up
Voglibose	7.5184	0.02035	−1.8284	Up
1,25-Dihydroxyvitamin D3 3-glycoside	4.4632	0.01656	−0.7329	Up
3,4,17-trihydroxy-9,10-seco-androsta-1,3,5(10)-triene-9-one	3.9242	0.02041	−1.0421	Up
N1,N8-Diacetylspermidine	2.43551	0.00131	−0.5018	Up
**control_16-vs.-GAD65_16**				
L-Alloisoleucine	14.6176	0.00695	1.14379	Up
3,4-Dihydro-2H-1-benzopyran-2-one	12.7497	0.03683	1.23439	Up
2-Acetyl-1,5,6,7-tetrahydro-6-hydroxy-7-(hydroxymethyl)-4H-azepine-4-one	8.03661	0.00385	0.94877	Up
2-C-Methyl-D-erythritol 4-phosphate	6.65553	0.02902	1.22807	Up
THTC	6.15843	0.00932	1.39183	Up
Dimetacrine tartrate	5.68664	0.04027	−1.2281	Down
6-HYDROXYANGOLENSIC ACID METHYL ESTER	2.2016	0.01755	−0.7035	Down
PC(DiMe(11,5)/MonoMe(11,3))	2.00352	0.0334	−2.1317	Down
Euphorbia factor Ti2	1.59097	0.03643	−1.7298	Down
5-Hydroxypentanoic acid	1.56521	0.01675	−0.7437	Down
**control_16-vs.-NZ9000_16**				
Phenethylamine glucuronide	4.48271	0.00479	1.89505	Up
MG(0:0/16:1(9Z)/0:0)	4.37551	0.02216	1.1632	Up
Deoxyinosine	4.10991	0.0031	1.26439	Up
Telbivudine	3.98936	0.03282	0.72069	Up
Ganoderic acid C_2_	3.76156	0.00152	1.50722	Up
(22R)-3alpha,7alpha,22-Trihydroxy-5beta-cholan-24-oic Acid	57.3482	0.0488	−1.5948	Down
3a,4b,12a-Trihydroxy-5b-cholanoic acid	42.7362	0.03138	−3.3462	Down
3α-Hydroxy-5β-chola-7,9(11)-dien-24-oic Acid	25.5582	0.02002	−2.4503	Down
5β-Chola-3,8(14),11-trien-24-oic Acid	19.5152	0.02147	−2.9188	Down
PI(16:0/18:0)	19.1612	0.04699	−5.4381	Down
**NZ9000_16-vs.-GAD65_16**				
N-Acetyl-D-glucosamine	8.9589	0.0309	0.65822	Up
Desglymidodrine	3.777	0.04995	0.53344	Up
Acetylcholine	3.43218	0.04823	0.42249	Up
Acipimox (5-methylpyrazine-2-carboxylic acid)	2.96428	0.00059	0.59656	Up
6-({3,5-dihydroxy-2-[hydroxy({2,3,4-trihydroxy-6-oxo-3-[3,4,5-trihydroxy-6-(hydroxymethyl)oxan-2-yl]cyclohexa-1,4-dien-1-yl})methyl]oxan-4-yl}oxy)-3,4,5-trihydroxyoxane-2-carboxylic acid	2.47561	0.02144	1.7766	Up
xi-2,3-Dihydro-2-oxo-1H-indole-3-acetic acid	9.93164	0.03713	−0.8193	Down
N1,N8-Diacetylspermidine	9.02402	0.01602	−1.0486	Down
Hippurin-1	7.81028	0.04452	−1.1046	Down
M 344	5.64693	0.02168	−1.4341	Down
(9Z,12Z,14E)-16-Hydroxy-9,12,14-octadecatrienoic acid	5.63666	0.04472	−0.4385	Down
**control_24-vs.-GAD65_24**				
Taurocholic acid	11.8384	0.03989	2.19745	Up
(9Z,12Z,14E)-16-Hydroxy-9,12,14-octadecatrienoic acid	8.46122	0.02868	1.77733	Up
(±)12(13)-EpOME	8.19732	0.00271	2.06938	Up
E,E-9-HpODE	6.12916	0.01663	2.53013	Up
LysoPC(0:0/16:0)	5.8749	0.02692	1.35126	Up
3-Amino-4,7-dihydroxy-8-methylcoumarin	15.4917	0.04592	−1.4871	Down
5-Aminopentanoic acid	9.89937	0.01249	−0.6199	Down
Gaboxadol	3.78973	0.004	−0.4838	Down
(3R)-3,4-Dihydroxy-3-(hydroxymethyl)butanenitrile 4-glucoside	3.45038	0.03324	−1.0679	Down
2-(2,4-dihydroxy-5-methoxyphenyl)-3-(3,7-dimethylocta-2,6-dien-1-yl)-5-hydroxy-8,8-dimethyl-4H,8H-pyrano[3,2-g]chromen-4-one	2.12585	0.01757	−1.4876	Down
**control_24-vs.-NZ9000**				
(9Z,12Z,14E)-16-Hydroxy-9,12,14-octadecatrienoic acid	9.65671	0.0021	1.92272	Up
(±)12(13)-EpOME	9.1705	0.00855	2.11728	Up
E,E-9-HpODE	6.76751	0.01541	2.40964	Up
Atrolactic acid	6.7476	0.00804	0.96461	Up
17-hydroxy-linolenic acid	6.48908	0.02538	1.87306	Up
7-Ketodeoxycholic acid	9.82503	0.01961	−0.6443	Down
Uric acid	3.27161	0.01999	−2.4394	Down
17R-HDHA	3.1493	0.0434	−0.8553	Down
Asymmetric dimethylarginine	2.71701	0.04101	−0.8598	Down
Allopregnanalone sulfate	2.60127	0.00377	−2.6514	Down
**NZ9000_24-vs.-GAD65_24**				
17-HOME(9Z)	5.92395	0.02213	0.83256	Up
(23S,24S)-17,23-Epoxy-24,29-dihydroxy-27-norlanost-8-ene-3,15-dione	5.85579	0.048	0.74105	Up
2-(3,4-dimethoxyphenyl)-5,6,7,8-tetrahydroxy-3-methoxy-3,4-dihydro-2H-1-benzopyran-4-one	5.43801	0.01118	0.66076	Up
Glycerol tributanoate	5.34647	0.00572	1.67588	Up
3,4-Dimethyl-5-pentyl-2-furanpentadecanoic acid	4.97022	0.04797	1.26745	Up
5-Aminopentanoic acid	10.9735	0.01238	−0.5518	Down
Voglibose	7.5184	0.02035	−1.8284	Down
1,25-Dihydroxyvitamin D3 3-glycoside	4.4632	0.01656	−0.7329	Down
3,4,17-trihydroxy-9,10-seco-androsta-1,3,5(10)-triene-9-one	3.9242	0.02041	−1.0421	Down
N1,N8-Diacetylspermidine	2.43551	0.00131	−0.5018	Down

## Data Availability

The original contributions presented in this study are included in the article/Supplementary Material. Further inquiries can be directed to the corresponding authors.

## Supplementary Materials

The following supporting information can be downloaded at: https://www.mdpi.com/article/10.3390/microorganisms14010176/s1. Figure S1. (A) The schematic map of oral immunization schedule of the NOD mouse. (B) The number of colonies of *L. lactis* in intestinal tract of NOD mice (n = 3) (* *p* < 0.05). Figure S2. Base peak chromatograms of 5 quality control samples in positive and negative ion modes. The *x*-axis represents Retention Time, while the *y*-axis represents Ion Intensity. Figure S3. Overview of the chromatographic separation (total ion chromatogram) and the extracted ion chromatogram for 5-hydroxypentanoic acid. Figure S4. Bar Chart of Up/Down-Regulated Metabolic Pathways from KEGG Enrichment Analysis. The *x*-axis represents the −log10 *p*-value for each pathway, and the *y*-axis shows the names of different pathways. The numbers on the bars indicate the count of differential metabolites annotated to the respective pathway, while the varying colors of the bars represent different KEGG pathway categories. Supplementary Tables S1: Raw data for Figure 2. Supplementary Tables S2: Raw data for Figure 3. Supplementary Tables S3: Raw data for Figure 4. Supplementary Tables S4: Raw data for Figure 5. Supplementary Tables S5: Data of detected metabolites. Supplementary Tables S6: Raw data for Figure 6. Supplementary Tables S7: Raw data for Figure S4. Supplementary Tables S8: Raw data for Figure 7.

## Abbreviations

The following abbreviations are used in this manuscript:

GAD65-*L. lactis*	recombinant Lactococcus lactis vaccine expressing GAD65
T1D	type 1 diabetes
16S rRNA sequencing	Integrated analyses of gut microbiota
DC (s)	dendritic cell (s)
Treg (s)	regulatory T cell (s)
SCFA (s)	short-chain fatty acid (s)
