# Supplementary figures and images for: Oral GAD65-L. lactis Vaccine Halts Diabetes Progression in NOD Mice by Orchestrating Gut Microbiota–Metabolite Crosstalk and Fostering Intestinal Immunoregulation
Source: Microorganisms. 2026 Jan 13;14(1):176. doi: 10.3390/microorganisms14010176 (PMC12844348; doi:10.3390/microorganisms14010176)

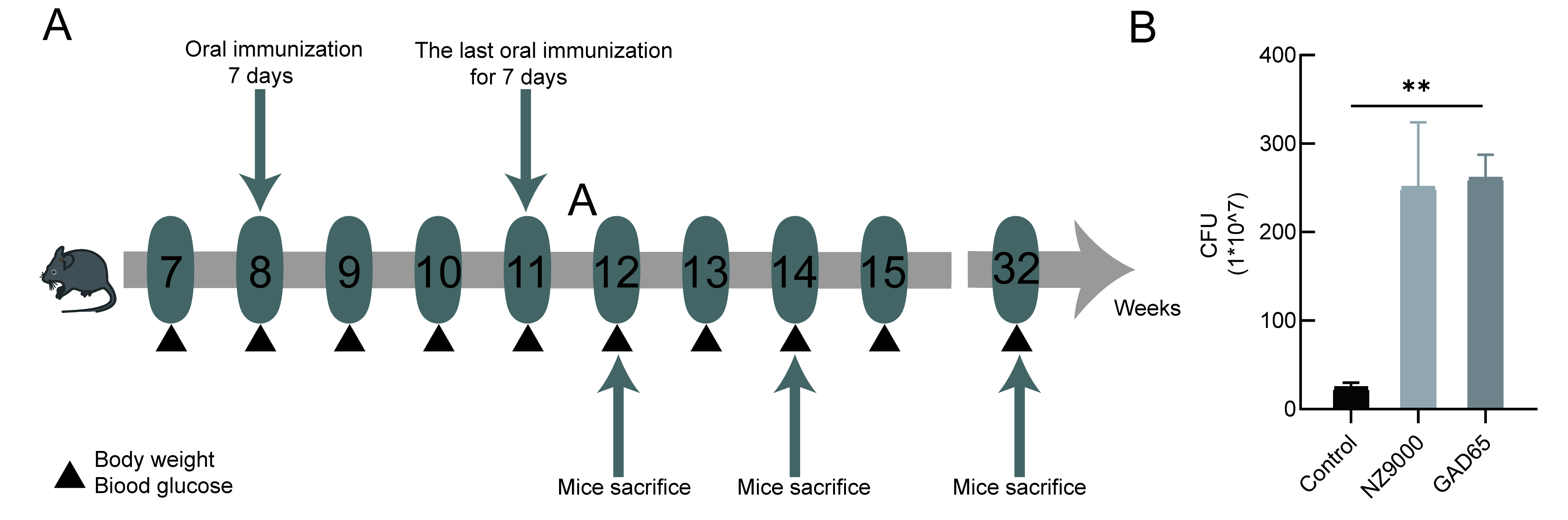

Supplement: Supplementary file 1 [file microorganisms-14-00176-s001.zip › Figure S1.tif]

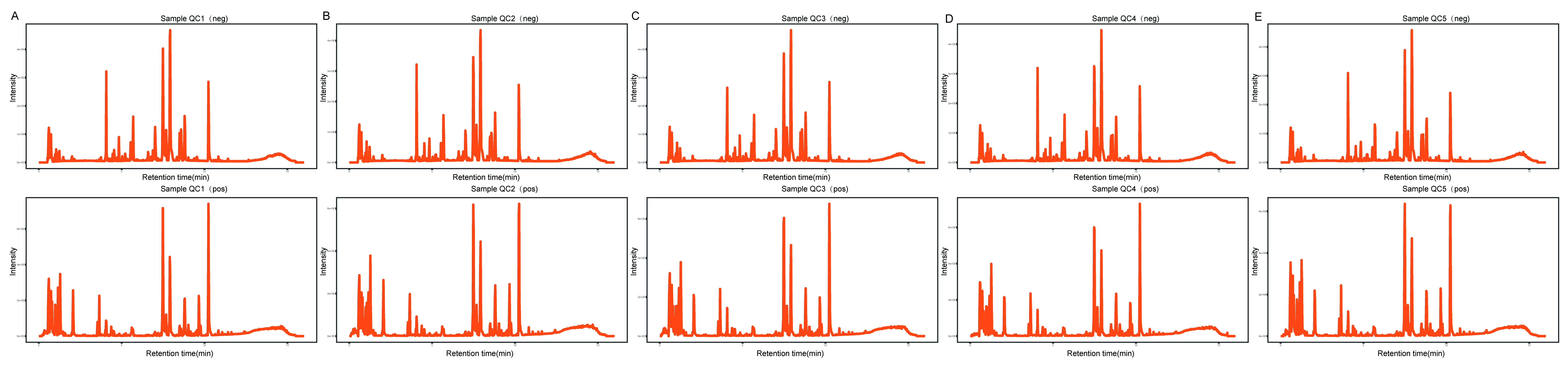

Supplement: Supplementary file 1 [file microorganisms-14-00176-s001.zip › Figure S2.tif]

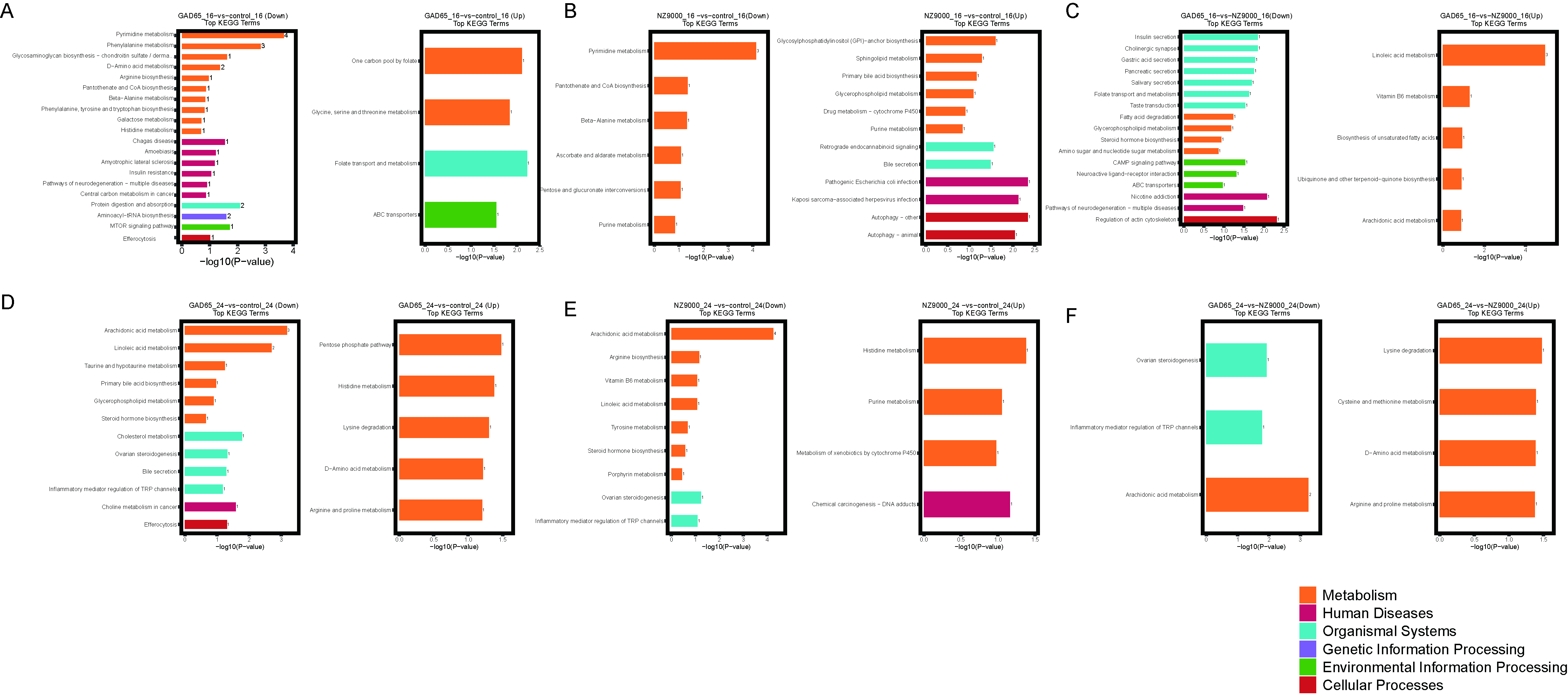

Supplement: Supplementary file 1 [file microorganisms-14-00176-s001.zip › Figure S4.tif]
